# Role of HLA Adaptation in HIV Evolution

**DOI:** 10.3389/fimmu.2015.00665

**Published:** 2016-01-18

**Authors:** Henrik N. Kløverpris, Alasdair Leslie, Philip Goulder

**Affiliations:** ^1^KwaZulu-Natal Research Institute for Tuberculosis and HIV, Nelson R Mandela School of Medicine, University of KwaZulu-Natal, Durban, South Africa; ^2^Department of Immunology and Microbiology, University of Copenhagen, Copenhagen, Denmark; ^3^HIV Pathogenesis Programme, Doris Duke Medical Research Institute, University of KwaZulu-Natal, Durban, South Africa; ^4^Department of Paediatrics, University of Oxford, Oxford, UK

**Keywords:** HIV-1, HLA class I, CD8^+^ T cells, viral fitness, viral adaptation, viral replicative capacity

## Abstract

Killing of HIV-infected cells by CD8^+^ T-cells imposes strong selection pressure on the virus toward escape. The HLA class I molecules that are successful in mediating some degree of control over the virus are those that tend to present epitopes in conserved regions of the proteome, such as in p24 Gag, in which escape also comes at a significant cost to viral replicative capacity (VRC). In some instances, compensatory mutations can fully correct for the fitness cost of such an escape variant; in others, correction is only partial. The consequences of these events within the HIV-infected host, and at the population level following transmission of escape variants, are discussed. The accumulation of escape mutants in populations over the course of the epidemic already shows instances of protective HLA molecules losing their impact, and in certain cases, a modest decline in HIV virulence in association with population-level increase in mutants that reduce VRC.

## Introduction

The ability of HIV to evade the immune response is one of the major challenges standing in the way of the development of a successful HIV vaccine. Although the drive to improve immune control via T-cell vaccines has been diminished following the Step and Phambili trials involving the MRKAd5 HIV-1 vaccine (1–4), success of a T-cell vaccine using a CMV vector in the SIV-macaque model (5–8) and the increasing recognition that CD8^+^ T-cells are likely to play a critical role in HIV eradication (9, 10) underlines the continuing importance of antiviral T-cell activity in HIV vaccine and Cure approaches. Understanding the impact of CD8^+^ T-cell escape both within the host and at the population level therefore remains of critical relevance to the field.

Almost 25 years have elapsed since the initial discovery that HIV variation could result in viral escape from the CD8^+^ T-cell response (11). This early work focused on the HLA-B*27-restricted response to an immunodominant epitope in the Gag protein KRWIILGLNK (“KK10”: Gag 263–272). Much has been learned about HIV adaptation to HLA class I-restricted CD8^+^ T-cell responses and its potential consequences from this single response. HLA-B*27 provides an especially clear example because, first of all, HLA-B*27 is strongly associated with slow progression to HIV disease (12–16). Second, the peptide-binding motif is so clean: only one amino acid is acceptable at position-2 (P2) in the epitope (17–20). Third, immunodominance is very strong (13), so, although a simplification, much of the story can be read by focusing on KK10 alone. Fourth, by chance, very few other CD8^+^ T-cell responses target epitopes in this region. Therefore, certainly in Caucasian populations infected with B clade virus, any mutations within KK10 can safely be assumed to have been the result of selection pressure driven by this HLA-B*27 response. Fifth, escape almost invariably involves substitution of Arg at P2, which effectively abrogates binding and immunogenicity of the epitope for any HLA-B*27-positive recipient of the variant. Finally, this immunodominant KK10 response illustrates an important feature of most, if not all the epitopes that appear to mediate HLA-associated control of HIV infection, namely that escape mutation within the epitope significantly reduces viral replicative capacity (VRC). This last point underlines that fact that viral fitness is a critical factor in determining timing and impact of escape mutations.

## Within Host HLA Adaptation: Lessons Taught by HLA-B*27

The HLA-B*27-restricted KK10 response was the first HIV-specific epitope described (21), a fact that is related to its immunodominance in HIV-infected individuals who express HLA-B*27 (13, 22). HLA-B*27 is reasonably prevalent in Caucasian populations (phenotype frequency ~8%) (20) and individuals expressing HLA-B*27 progress relatively slowly to disease (12–14, 23, 24); therefore, longitudinal studies of this response were feasible. The KK10 escape mutations within KK10 appeared to be selected late – after a decade or more of infection – and were associated with progression to AIDS (13). The dominant observed escape mutation, R264K, arises at the anchor position-2 (P2) in the epitope that is believed to require Arginine for adequate binding to HLA-B*27 (17–20). These data suggested the possibility that KK10 might be an important immune response mediating the protection against rapid disease progression linked with HLA-B*27.

The main features of this “B27 story” have remained broadly unaltered over the past two decades, although it has become more complex. First, KK10 is by no means the only HIV-specific response, nor is it necessarily the first CD8^+^ T-cell response to emerge in the very early phase of infection in subjects expressing HLA-B*27. The initial response appears to be against the Vpr epitope “VL9” (Vpr 31–39: VRHFPRIWL) and escape in VL9, again via an Arg → Lys substitution at the P2 anchor, predates the R264K escape mutation within KK10. Although KK10 is usually the dominant response in chronic infection, more sensitive and more comprehensive assays have revealed between 15 and 20 additional HIV-specific HLA-B*27-restricted epitopes (22; Leitman, Personal communication). This includes a Pol epitope “KY9” (Pol 901–909, KRKGGIGGY) which in some subjects can be the immunodominant response and within which selection of escape mutations (Arg → Lys at P2 and/or Gly → Glu at P8) can predate escape within KK10 (22; Leitman, Personal communication) (Table 1). Thus, the HLA-B*27-restricted CD8^+^ T-cell response is much more broadly based than initially realized.

**Table 1:** **Selected key epitopes with HLA restriction, and associated HIV-1 escape and compensatory mutations**.

Protein	Epitope	HLA restriction	Sequence^a^	Escape mutation	Compensatory mutation
Gag	TL9	B*07:02/42:01/81:01	TP**Q**DLN**T**ML	Q182T, Q182E/G/S, T186S	E177D, V191I
Gag	GL9	B*07:02	GP**S**HKARVL	S357G	
Gag	RM9	B*07:02/42:01	RPGGKK**H**Y**M**	H28Q/S/R, M30R/K	R20S
Gag	KK10	B*27:05	K**R**WII**L**GLNK	R264K/G/T/Q, L268M/I	S165N, S173A
Gag	QK10	B*27:05	Q**R**GN**F**RNQRK	R380K, F383Y	
Pol	KY9	B*27:05	K**RK**GGIG**G**Y	R902K, K903R, G908E	
Nef	WF9	B*27:05	W**R**FDS**R**LAF	R104K, R108X	
Vpr	VL9	B*27:05	V**R**HFP**R**IWL	R32K, R36G	
Vif	KK11	B*27:05	K**R**KPPLPSVTK	R159G	
Gag	NY10	B*35:01	NPPIPVG**D**IY	D260E	
Pol	TI8	B*51:01	TAFTIPS**I**	I135T/R	
Gag	ISW9	B*57:02/57:03	**AI**SPRTLNAW	A146P, I147L/M	
Gag	KF11	B*57:01/57:03	K**A**F**S**PEVIPMF	A163G/S	S165K/N
Gag	TW10	B*57:03/58:01	TS**T**LQEQIAW	T242N	H219Q, I223V, M228L
Gag	QW9	B*57:02/57:03/58:01	Q**AT**QDVKNW	A309S, T310S	
Nef	KF9	B*57:02/57:03/58:01	K**A**AFDLSFF	A83G	
Env	QL11	B*58:02	QTRVLAERYL	None	

*^a^Bold residue indicates escape mutation selected*.

Second, although selection of the R264K is associated with progression to AIDS, the mutation L268X (where X represents Met or Ile at P6 in the KK10 epitope) occurs prior to R264K, and this is associated with reduced immune control. Typically, progression to a CD4 count of <200 cell/mm^3^ or AIDS appears to require the mutation at Arg-264 which abrogates binding of KK10 to HLA-B*27, and therefore would abolish the CD8^+^ T-cell response altogether, whereas L268X merely reduces recognition. A subset of the KK10-specific CD8^+^ T-cells that recognize the wild-type epitope have a high functional avidity and are the most potent inhibitors of viral replication (25–30); so loss of this subset of CD8^+^ T-cells as a result of selection of the L268X mutant loosens the hold of the KK10 response over the virus. As described further below, this now appears to prepare the conditions for R264K to be selected.

Third, the selection of R264K many years after infection prompts the question of why this occurs so late. Part of the explanation is that the R264K mutant itself inflicts a substantial cost on VRC such that a compensatory mutation – S173A – needs to be selected at the same time as the escape variant (23, 31). In fact, alternative escape mutants to R264K are also selected, principally R264G and R264T, that do not impair VRC as much as R264K (32), but impair VRC substantially more than the R264K/S173A combination. Thus, there is a balance between the benefit the virus accrues from evading the KK10 response and the cost of the escape mutant. Selection of R264K invariably follows that of L268X, which, as described above, weakens the efficacy of the KK10 response; in the setting of L268X, therefore, the additional benefit of R264T and T264G may not be of sufficient value to the virus to be worth the cost. On the other hand, in the setting of a highly potent KK10 response, prior to the selection of L268X, R264T, or R264G might result in the complete evasion of the KK10 response, at a cost to VRC that was of overall benefit to the virus. For these reasons, with rare exceptions, R264K escape is only seen in combination with S173A, following selection of L268X (31), whereas R264T or R264G are typically selected without L268X having been selected previously (23) (Table 1).

It should be added here that late escape does not always imply a strong CTL response and a fitness cost resulting from the escape mutation. Late escape could also arise in the setting where either there are constraints against escape mutation or the CTL response is weak.

The fourth complication is the influence of the particular viral context within which these escape mutants are selected. This is most readily observed when considering the escape pathways available to HIV from different clades of virus. The options for KK10 escape in B clade infection have been described above. In C clade virus infections, which predominate in Africa and therefore in the global epidemic, the situation differs. In particular, instead of Ser at Gag-173, the C clade consensus residue at this position is Thr. This clade-specific difference at Gag-173 might be expected to influence the cost of R264K escape and the necessity for a compensatory mutation at Gag-173 as is observed in B clade infection. In fact, what we see in KK10 escape in C clade HLA-B*27-positive individuals is the selection of R264K without prior escape at L268, and the compensatory mutation is typically S165N (Brener, Personal communication). In addition, changes in residues within the cyclophilin A-binding loop, a region within the capsid protein spanning Pro-217 to Pro-225 (PVHAGPIAP), may provide an alternative route by which compensation for the fitness cost of R264K can be achieved in C clade infection. However, the R264K/S173A escape and compensatory pathway employed predictably in B clade infection is not adopted in C clade. These examples illustrate the point that the escape pathways adopted in each infected individual are shaped by the particular autologous viral sequences in operation as well as by the immune response.

The final complication to add to the original “B27 story” is the question of what distinguishes the HLA-B*27-positive HIV-infected individuals who are “elite controllers” [antiretroviral therapy (ART)-naïve subjects whose viral loads are undetectable (<50 copies/ml plasma)] from those who do not exhibit the same degree of viremic control. In some cases, as described above, if KK10 escape mutants have been selected, this would explain failure of the KK10 response to suppress viral replication. In other subjects, in whom KK10 escape mutants have not been selected, qualitative differences in the ability of KK10-specific CD8^+^ T-cells to recognize low concentrations of the KK10 variants may help to explain elite control in HLA-B*27-positive individuals (33). Although Arg at P2 is believed to be a requirement of any HLA-B*27-binding peptide, it is possible that an Arg → Lys mutant might merely reduce presentation of the peptide, as opposed to abolishing it altogether, which might be sufficient for some KK10-specific cells of very high functional avidity to maintain recognition of, and kill, even those virus-infected cells expressing the KK10 variant.

## Within Host HLA Adaptation: Lessons Learnt from Non-HLA-B*27 Alleles

### HLA-B*57 and HLA-B*58:01

HLA-B*57 is highly enriched in HIV elite controllers [reviewed in Ref. (34)] and is the HLA class I allele with the strongest impact on viral load setpoint and disease progression (12, 15, 16, 35–40). Within the sub-Saharan African population, where the HIV epidemic is concentrated, HLA-B*57:03 is the most prevalent HLA-B*57 subtype, expressed in ~4–5% of individuals. It differs by one amino acid from HLA-B*57:02 (Leu-156 in B*57:03, Arg-156 in HLA-B*57:02), the less common HLA-B*57 subtype found in sub-Saharan African populations. The HLA-B*57 alleles are also closely related to HLA-B*58:01, all being protective against HIV disease progression and sharing presentation of many of the same epitopes. The hierarchy of protection is, in ascending order, HLA-B*58:01, HLA-B*57:02, and HLA-B*57:03 (41), and this order is associated with the number of p24 Gag-specific CD8^+^ T-cell responses that drive selection pressure on the virus. In the case of HLA-B*57:03, selection of mutations arises in three p24 Gag-specific epitopes, in a predictable sequence (42–44). HLA-B*57:02- and HLA-B*58:01-restricted p24 Gag responses drive selection pressure in only two instances.

The TW10 epitope (TSTQEQIAW, Gag 240–249), is presented by all three closely related HLA-B*57:02/57:03/58:01 class I molecules, and escape mutants at T242X are selected in 70–90% of individuals expressing these alleles. This mutation shares many features with the R264K mutation within the HLA-B*27-restricted KK10 epitope described above. The most common mutant, T242N, effectively abrogates recognition by CD8^+^ T-cells (42, 45, 46). At the same time, T242N also reduces VRC (42, 43, 45). Unlike R264K, reversion of T242N following transmission to a recipient who does not express the relevant HLA class I molecule, does arise commonly (42), because compensatory mutants appear to be less efficient at correcting the fitness cost than S173A is for R264K (32). However, if compensatory mutations, such as H219Q, I223X, and M228X, are selected in combination with T242N, the speed of reversion post-transmission may be considerably diminished (47) (Table 1).

The p24 Gag epitope that is uniquely presented by HLA-B*57:03 among this group of related HLA-B*57/58:01 alleles is KF11 (KAFSPEVIPMF, Gag 162–172) (35). This is the immunodominant HLA-B*57:03-restricted epitope in chronic infection, targeted by >75% of infected individuals expressing HLA-B*57:03. The sequence of events observed for escape within KF11 has close similarities with those described above for KK10 escape through R264K. The initial escape mutation within KF11 is A163X, typically A163G, located at position-2 in the epitope, which reduces but does not abrogate recognition of the epitope by KF11-specific CD8^+^ T-cells. This mutant carries a cost for the virus, in that A163G reduces VRC (48, 49). The fitness cost of A163G is substantially, although not fully, restored by the compensatory mutation S165N that emerges subsequent to A163G (43, 48, 49). Furthermore, the combination of A163G and S165N results in the complete loss of recognition of the epitope by CD8^+^ T-cells. Thus, as with the fully compensated HLA-B*27-R264K escape, the virus ultimately achieves almost the perfect solution, that is, complete escape from T-cell recognition with minimal loss of VRC (Table 1).

In summary, the HLA-B*57/58:01 alleles present a clear picture, consistent with that described in relation to the HLA-B*27-restricted Gag response, suggesting a mechanism for HLA-linked control of HIV through the dominant targeting of Gag, with strong selection pressure for escape variants that reduce VRC and that therefore require the virus also to select compensatory mutants for adequate replication. However, although individual compensatory mutants, such as S165N, may help to compensate for the fitness cost of the escape mutant, in this case A163G, an accumulation of costly escape mutants may severely attenuate the virus (43). This is what appears to result in the face of the three HLA-B*57:03-restricted, p24 Gag-specific CD8^+^ T-cell responses that drive selection pressure on HIV. Thus, although the virus is constantly generating variants that may diminish the cost of escape mutants, in many cases, the compensation will be incomplete, and/or take some time, and therefore a broad Gag-specific CD8^+^ T-cell response is likely to be highly effective in long-term suppression of viremia. This may also explain the observation that HLA-B*57-positive elite controllers have often selected rare B*57 mutations that substantially reduce VRC with no apparent accompanying compensatory variant (50). By contrast, escape mutations outside Gag, such as in Env, may have minimal impact on VRC (51), and these responses are typically not associated with control of viremia (52).

Additional insight into the effect of MHC class I alleles involved in control HIV infection comes from an elegant study by Wroblewski et al. that over a period of 50 years followed three communities of chimpanzees (*P. t. schweinfurthii*) infected with SIVcpz (53) in the Gombe National Park, Tanzania (54). DNA and RNA were extracted from fecal dumps to study longitudinal changes of MHC and SIVcpz viral loads over time. Three chimpanzee MHC alleles (Patr-B*06:03/B*22:03/B*22:05) were enriched in the SIVcpz-infected groups. Of particular interest, in the case of HLA-B*57 and control of HIV, is the Patr-B*06:03 allele because it phylogenetically clusters with HLA-B*57:01, shares a similar B-pocket binding structure and is associated with lower SIVcpz viral load. SIVcpz sequencing showed variation in Gag-242, T242S, suggesting selection pressure from Patr-B*06:03-restricted SIVcpz specific CD8^+^ T-cells and similar to that demonstrated for the HLA-B*57/58:01-associated T242N mutation in HIV. The study from Wroblewski et al. also suggests that SIVcpz infection results in particular Patr-B alleles becoming enriched in the chimpanzee population.

### Other African HLA-B Alleles Contributing to Control

The other HLA allele strongly associated with protection against disease progression in African subjects infected with HIV is HLA-B*81:01 (16, 38, 55, 56). HLA-B*81:01 restricts a dominant TL9 p24 Gag response (TPQDLNTML, Gag 180–188) (57) that drives, in particular, the selection of T186S (44, 58, 59) that substantially reduces VRC. The compensatory mutations at Gag-190 only partially restore the fitness cost from these intra-epitope mutations (60).

This single p24 Gag epitope, TL9, has taught us several additional aspects of CD8^+^ T-cell immunity. This is the most targeted HIV-specific epitope in sub-Saharan Africa, being presented by several highly prevalent HLA alleles, including HLA-B*07:02/B*39:10/B*42:01/B*81:01 and HLA-C*08:02. The immunodominance pattern for each HLA allele is influenced by TCR bias, such that the TCR gene rearrangements necessary for the immunodominant TL9-specific responses arise more readily than those necessary for non-dominantly targeted epitopes restricted by the same allele (61). A further point this epitope illustrates is that although ultiple HLA-B alleles present TL9, the frequency and patterns of the mutations they select are often distinct, and these have a different impact on VRC. Several explanations have been proposed to explain the stronger selection imposed via HLA-B*81:01 on TL9 compared to HLA-B*42:01. First, TL9-specific CD8^+^ T-cells restricted by HLA-B*81:01 and HLA-B*42:01 have different TCR clonotype usage. Second different TCR affinities may result in different cross-recognition properties of TL9 variants (62, 63). Third, the HLA-specific conformation of the TL9 peptide differs greatly in 3-dimentional space (59). These factors may explain a wider phenomenon of distinct escape pathways adopted by the virus in the face of CD8^+^ T-cell responses to the identical epitope but restricted by different, albeit closely related HLA class I molecules. At the population level, this can lead to discordant HLA-associated impact on VRC and on disease outcomes (44).

HLA-B*42:01 and the closely related HLA-B*42:02 make a particularly informative case study, as they differ by only a single amino acid (64), occur almost exclusively on the same haplotype together with HLA-A*30:01 and HLA-C*17:01, and, as Bw6 alleles, they have no interaction with KIR ligands (65). Thus, disease associations can be more easily attributed to the CD8^+^ T-cell responses these alleles, respectively, generate. The impact of the single residue change is striking, with HLA-B*42:01 significantly superior to HLA-B*42:02 as a protective allele (55, 58). Perhaps unsurprisingly, the ultimate mechanism reflects the patterns observed for HLA-B*27 and HLA-B*57, in that HLA-B*42:02 has a narrow Gag response and does not effectively present key Gag epitopes, such as p17 Gag RM9 and p24 Gag TL9, that are strongly targeted by B*42:01 (64). This is consistent with previous studies demonstrating differences in rates of AIDS progression associated with single amino acid differences between HLA-B*35 subtypes, such as B*35:01 compared with B*35:02/03 (66). Again these differences in disease outcome are linked to higher frequency of Gag epitope targeting by HLA-B*35:01 compared with HLA-B*35:02/3 (67).

### Disease-Susceptible HLA Alleles

Insight to the mechanisms underlying HIV control also comes from studies of HLA alleles associated with rapid progression. In contrast to protective alleles such as HLA-B*27/57/58:01/81:01, the CD8^+^ T-cell response restricted by disease-susceptible HLA fail to constrain viral replication and hence do not impose selection pressure on the virus (68). HLA-B*42:02, as described above, is an example of a disease-susceptible allele. The second example is HLA-B*58:02 that is associated with rapid disease progression (38, 55, 69), despite being closely related to a protective allele, HLA-B*5801. These two HLA molecules differ by only three amino acids, but, as with B*42:01 and B*42:02, the consequence is two very distinct immonodominance patterns. The dominant HLA-B*58:01-restricted response is the p24 Gag epitope TW10, escape from which reduces VRC (described above). However, the dominant HLA-B*58:02-restricted response is directed toward an epitope in Env, targeting of which is associated with high viral loads and no selection pressure (69). These observations are consistent with a recent study that identifies 21 epitopes with disease progression, of which only one is located in p24 Gag and the majority (15 epitopes) arise within Env or Pol (70).

The question of what happens when an individual expresses both a protective and disease-susceptible allele has been addressed by Leslie et al. (55) (JV 2010) who showed that the effect of alleles on immune control is additive. Viral loads are intermediate. CTL responses are not dominated by Gag, as in subjects only expressing protective alleles, but include responses in Env and in the accessory/regulatory proteins (52).

### HLA-C Contribution to HIV Control

Although HLA-C is expressed at lower levels than either HLA-A or HLA-B on the cell surface (71), a single nucleotide polymorphism (SNP), arising 35 kilobases upstream of the HLA-C locus, was shown to be protective against HIV disease progression (15, 16). Subsequently, this polymorphism was associated with higher HLA-C expression levels on the surface of T-cells (40) independent of HLA-A and HLA-B linkage disequilibrium (72). The mechanism is linked to variation within the 3′ untranslated region of HLA-C that regulates the binding of the microRNA Has-miR-148a (expressed on chromosome 7) and thereby also the levels of HLA-C expression (73). Variation in the gene encoding the microRNA Hsa-miR-148a directly affects HLA-C allele expression, such that high expression of miR-148a down-regulates those HLA-C alleles containing the corresponding microRNA-binding site (74). These studies suggest a new mechanism in which differential expression of HLA-C alleles may play important roles in control of viremia due to greater HLA-C restricted CD8^+^ T-cell activity and selection pressure (38, 52, 72). Thus, differences in relative HLA expression levels (71) may directly impact on CD8^+^ T-cell activity and contribution to HIV control.

## Population-Level HLA Adaptation

### HLA Footprints

**Figure 1:**
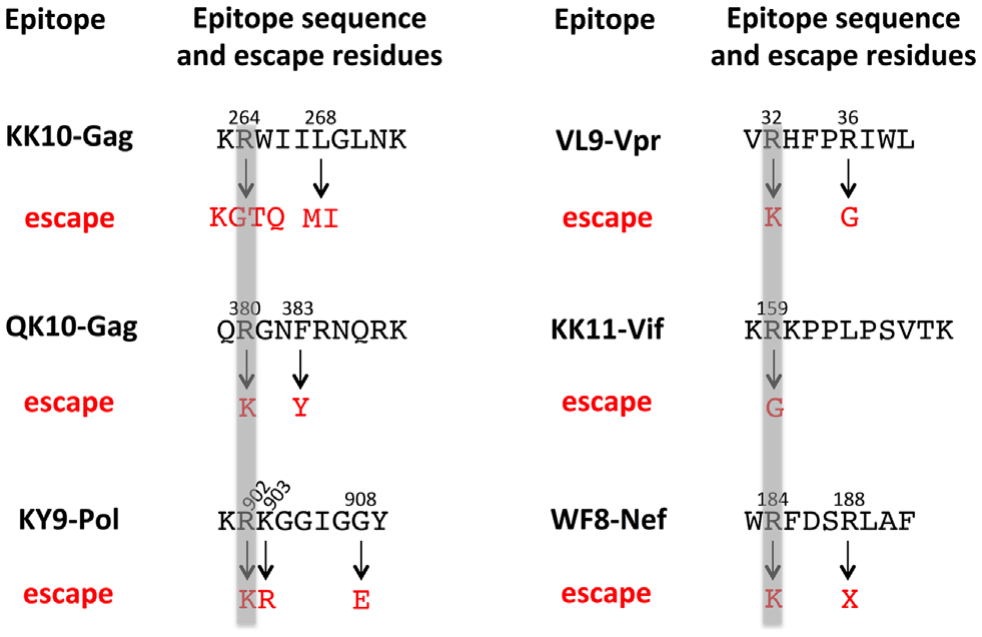
HLA-B*27 footprints in B clade HIV show a predictable pattern of escape mutation among six different epitopes. The 12 HLA-B*27-associated escape variants within six HLA-B*27-restricted epitopes are shown in red. Shaded are the common escape positions at P2 in the epitope in each case, in all cases from Arg → Lys or Gly. Data from Carlson et al. (44, 75).

As mentioned above, escape mutations within HLA-B*27-restricted epitopes in addition to the immunodominant Gag epitope KK10 are selected in HIV-infected individuals expressing this allele. These can be identified in cohort studies of HLA-typed subjects as HLA-B*27 “footprints” on the HIV sequence via demonstration of statistical associations between the presence of a variant, such as R264X (where X is either K, G, T, or Q), and expression of HLA-B*27. Carlson et al. (75) have constructed an escape map of the HIV B clade proteome, from the study of 1888 ART-naïve, chronically infected individuals. A similar escape map has been constructed for Gag, Pol, and Nef C clade sequences (76). From these studies, 12 HLA-B*27-associated mutations can be mapped onto six HLA-B*27-restricted CD8^+^ T-cell epitopes (Figure 1). A clear pattern can be identified from observation of these epitopes and the escape mutants that arise therein. In accordance with the requirement for Arg at P2 in HLA-B*27-binding peptides, all these epitopes carry Arg at P2, and in all 6 epitopes, substitution of Arg at P2 is the common (although not exclusive) escape route for the virus.

The realization that the viral sequencing of large cohorts of HLA-typed individuals could reveal effectively all the escape mutants selected at any meaningful level was first described by Moore et al. (77). These types of analyses are particularly valuable in identifying responses that are functional, as only these will impose sufficient selection pressure on the virus for escape mutants to be selected. The identification of HLA footprints can also help to uncover previously unknown epitopes that might make critical contributions to the acute response but are often undetectable in chronic infection, following early escape in acute infection.

It is also important to note that the HLA footprints can be “indirect” (76): that is, they are dependent on the presence of another HLA footprint: an example here would be the compensatory mutant S173A, whose selection in HLA-B*27-positive individuals is dependent on coselection of the R264K escape mutant. Additionally, there may be HLA footprints that are not necessarily linked with viral escape from the CD8^+^ T-cell response, but may be selected because they affect KIR binding to peptide–MHC complexes and thereby influence NK-mediated killing (78).

The final point concerning the identification of HLA footprints on the viral proteome is the information that these studies provide about which responses are important for immune control. In the study of the C clade epidemic in South Africa, it emerged that the number of Gag footprints per HLA molecule was strongly linked to the HLA-associated viral setpoint (68). So, highly protective HLA molecules, such as HLA-B*57/58:01/81:01, select a large number of Gag escape mutants, whereas disease-susceptible alleles, such as HLA-B*18:01/58:02, select none. This relationship did not apply to the number of Pol or Nef HLA footprints. Furthermore, the inverse correlation between viral load and number of HLA footprints was the strongest when limiting the analysis only to reverting mutants, that is, mutants that tend to revert back to the original sequence following transmission to an HLA-mismatched recipient (discussed further below). Following on from studies showing strong correlations between the breadth of the Gag CD8^+^ T-cell response and viral suppression, and inverse correlations for Nef- and Env-specific CD8^+^ T-cell responses (52), these data provide an entirely independent piece of evidence supporting the central role of Gag-specific CD8^+^ T-cell responses in the control of HIV infection.

### Transmission, Persistence, or Reversion of CTL Escape Variants

The first question that arises is whether the CD8^+^ T-cell escape mutants can be transmitted. A number of mother–child transmission studies (79–81) and adult horizontal transmission studies (43, 76, 80, 82–85) have shown that this is indeed the case. Interestingly, a recent analysis of adult transmission (76) showed that where a mixture of wild-type and variant sequences is present in the donor, the wild-type virus is preferentially transmitted. This, in itself, is further supporting evidence that some of these escape mutants have a real impact on viral fitness. Having said this, it is clear that even escape mutants that reduce VRC significantly can be transmitted, and often are (43, 47, 76, 80–85).

The next question is whether transmitted variants would persist in HLA-mismatched recipients or revert back to the original sequence in the absence of the CD8^+^ T-cell response that drove their selection. In theory, one might anticipate that variants having effectively no impact on VRC would persist, while those reducing VRC would revert. In the case of the HLA-B*27-associated R264K mutant, the fitness cost of this escape mutation is almost entirely corrected by the simultaneous selection of the compensatory S173A variant (31). Thus, transmission of the R264K/S173A combination to an HLA-B*27-negative recipient does not appear to result in reversion back to wild type (32). Escape mutants that significantly reduce VRC, and for which compensatory mutants only partially recover the fitness cost – examples described above include the HLA-B*57/58:01-associated mutant T242N, or the HLA-B*81:01-associated T186S (43, 48, 49, 51, 60) – do revert the following transmission to HLA-mismatched recipients. However, reversion does not necessarily happen within a few months of transmission in spite of the peak viral loads of ~10^7^ copies/ml plasma that occur during acute infection; rather, reversion may not occur for some years (42, 47, 80). This suggests that in some cases, the fitness costs of these mutants may be mitigated by an unpredictable combination and variety of compensatory mutants, the precise nature of which may vary from patient to patient and therefore that may be difficult to pinpoint. Alternatively, if viral loads are high and immune responses are weak, selection pressure may not be sufficient to discern between viruses with modestly differing VRC. Put another way, the selection pressure for reversion may be relatively weak in such settings, whereas the original pressure for the selection of the escape mutant must have exceeded the cost to VRC.

There is some evidence in support of this latter hypothesis from studies of infected children. In contrast to adult infection, in which a vigorous immune response brings viral load to a setpoint within 6 weeks of infection, viral loads in ART-naïve children remain at >1 × 10^6^ copies/ml for the first year or more and decline slowly to a quasi-setpoint at around 5 years of age (86, 87). This gradual decline in viremia in childhood infection coincides with the development of a more effective CD8^+^ T-cell response with increasing age (88), and it appears that reversion of mutants, such as T242N, in HLA-B*57/58:01-negative children is delayed until the maturing antiviral immune response is sufficiently potent to impose significant traction on the virus (80).

### Impact of Transmission of Escape Variants: HLA-Matched Recipients

**Figure 2:**
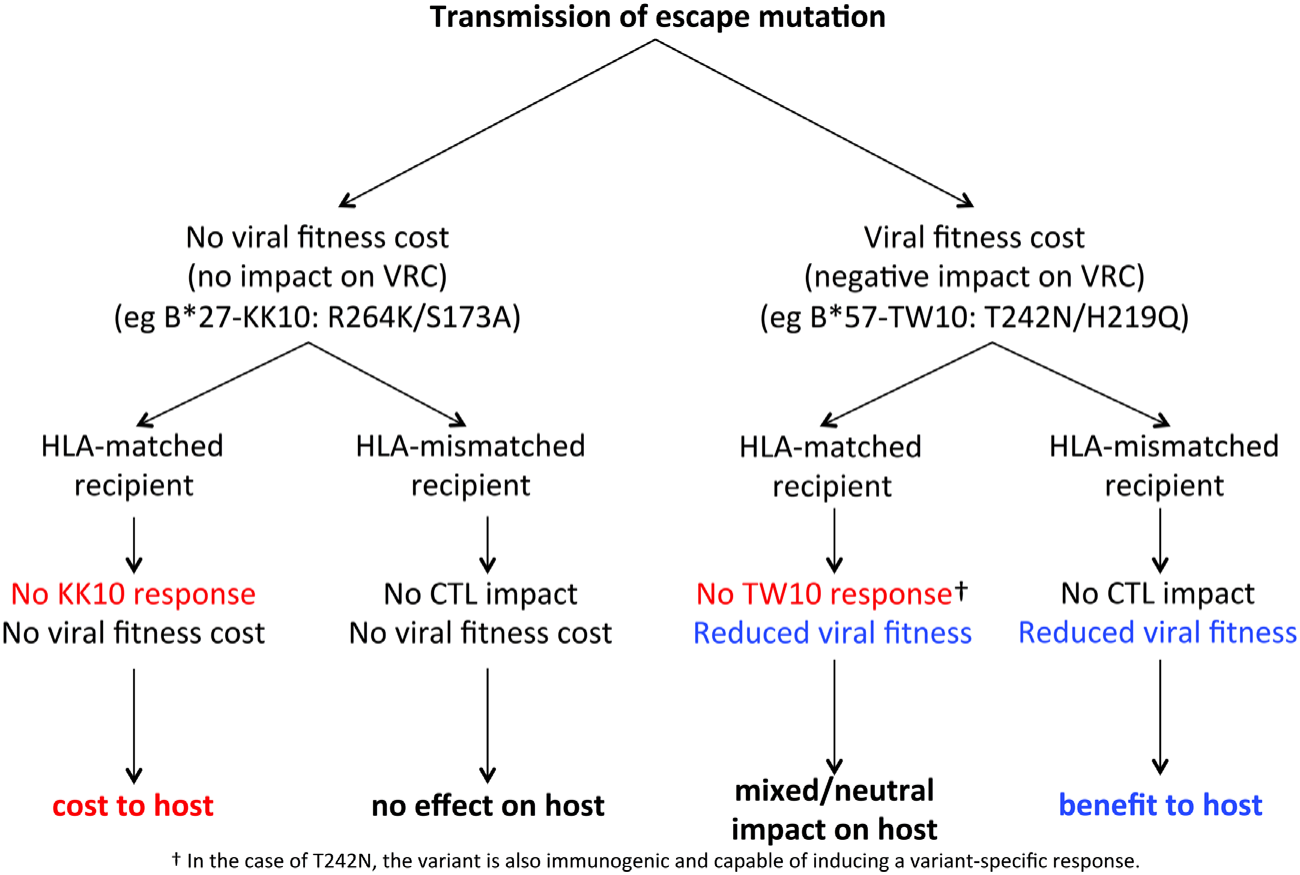
**Impact of transmission of escape mutant to HLA-matched and to HLA-mismatched recipient**. Two examples are shown to illustrate differing impact of transmission of escape variants. Left: transmission of escape mutation (R264K) where the virus is fully compensated by the compensatory mutation S173A. Right: transmission of escape mutation (T242N), where the compensatory mutant H219Q does not fully restore viral fitness (H219Q).

We establish above the fact that HLA-B*27-driven escape mutants typically include variants that abrogate HLA binding of the variant peptide that can be transmitted, and that, in the case of epitopes such as KK10, these variants do not normally revert post-transmission. One would predict, therefore, that the HLA-B*27-positive recipients of these variants would fail to generate any of the responses that would be expected to drive selection pressure on the virus (Figure 2). The impact of escape-variant transmission to HLA-B*27-positive adults has not been reported to date. However, in a small study of six HLA-B*27-positive, HIV-infected children, a KK10 response was not detectable in the children who were recipients of R264X variants from the mother, and progression was relatively rapid. By contrast, when wild-type KK10-encoding virus was transmitted, a strong KK10 response was evident. Indeed, one such child was a very rare pediatric “elite controller,” with viremia remaining below the limit of detection (<50 c/ml) for almost a decade. Progression finally occurred following the selection of the R264T mutant and loss of the KK10 response (89).

The outcome of escape variant transmission would differ from the scenario described for KK10 above if the escape mutant did have a significant fitness cost. An example here is the HLA-B*57/58:01-associated escape mutant T242N, described above, commonly driven by the TW10 response restricted by these alleles (Figure 2). Like R264K, T242N is a variant that significantly reduces VRC; however, S173A almost perfectly repairs the damage caused by R264K, the H219Q/I223V/M228L combination of compensatory mutations for T242N incompletely restores VRC (32). The fitness cost of T242N does vary quite considerably between individuals (90), depending upon the overall Gag sequence context in which it sits, and the timing of reversion of T242N back to wild type can range from months to years following transmission to an HLA-B*57/58:01-negative recipient (47). Therefore, the consequence for HLA-B*57/58:01-positive recipients of T242N escape variants is mixed: on the one hand, the virus has lower VRC and this is therefore a benefit; on the other hand, the recipient is deprived of the TW10-specific response. A further complication here is that the T242N variant does not prevent binding of TW10 to HLA-B*57/58:01, and a T242N-specific response can be generated (91). However, the efficacy of variant-specific responses, such as these, is not well established as the variants are often less immunogenic than the wild-type (92). The precise cost/benefit balance to the newly infected recipient might hinge on the degree of compensation achieved by the virus to recover the fitness cost of the escape mutant and the potency of the CD8^+^ T-cell response that is nullified by transmission of the escape variant.

### Impact of Transmission of Escape Variants: HLA-Mismatched Recipients

In the situation where these two respective mutants are transmitted to HLA-mismatched recipients, the effects would be nil in the case of the fully compensated R264K variant and of benefit to the recipient in the case of the T242N variant that still carries a fitness cost (Figure 2). This process, then, of transmission of escape mutants would only be detrimental in the minority of cases where the HLA of the recipient happened to match that of the donor. This result is precisely that described in studies of Zambian adult transmission pairs (82, 84) showing that newly infected individuals fared better in terms of CD4 count and viral setpoint if the virus they were infected with carried escape mutants associated with protective HLA-B alleles such as HLA-B*57/58:01/81:01, unless the new recipients themselves carried those same protective alleles as the donor. In other cases, all things being equal, transmission of virus with low VRC results in higher CD4 counts and lower viral loads in the recipient.

### Accumulation of Escape Variants at the Population Level

The observation that escape mutants with effectively no fitness cost attached could be transmitted and persist long term in HLA-mismatched recipients would suggest that over time, the frequency of these escape variants would increase and eventually become the dominant form. An indication that this process might indeed be occurring arose from the statistical data describing associations between certain HIV amino acid polymorphisms and expression of particular HLA class I molecules, as described above (75). The majority of these associations are between the HLA molecule and the minor variant – for example, R264X (where X is K/G/T/Q) is found in ~10–20% of HLA-B*27-positive individuals and in 5–10% of HLA-B*27-negative subjects (47). However, a small number of these HLA associations with HIV amino acid polymorphisms show a negative association between the relevant HLA molecule and the minor variant, and thus the HLA association is with the consensus sequence. One example is the HLA-B*27 association with an Arg → Lys substitution at Nef-184 (Figure 1). In fact, the consensus sequence has Lys at Nef-184. One possible explanation for such a negative HLA association with the consensus residue would be that this escape mutant has accumulated over time to replace the original “wild-type” residue. Such epitopes have been termed “negatopes” (93), both for the reason that they are epitopes containing a residue negatively associated with the restricting HLA molecule and because a peptide synthesized according to consensus sequence would carry the escape mutant, and therefore even if a CD8^+^ T-cell response existed, it would almost by definition not be detected. The first example was the Nef epitope KAAFDLSFF (Nef residues 82–90), which is restricted by the closely related HLA molecules HLA-B*57 and HLA-B*58:01. The escape mutation is the A → G substitution at P2 in the epitope, which substantially reduced binding of the variant to HLA-B*57/58:01. In C clade infection, the A83G variant is in fact present in >50% of sequences overall. Thus, peptides synthesized based on the consensus would be testing recognition of the escape mutant KGAFDLSFF that does not bind efficiently to HLA-B*57/58:01, and therefore no response would be detected even when a strong response to the wild-type KAAFDLSFF was present.

**Figure 3:**
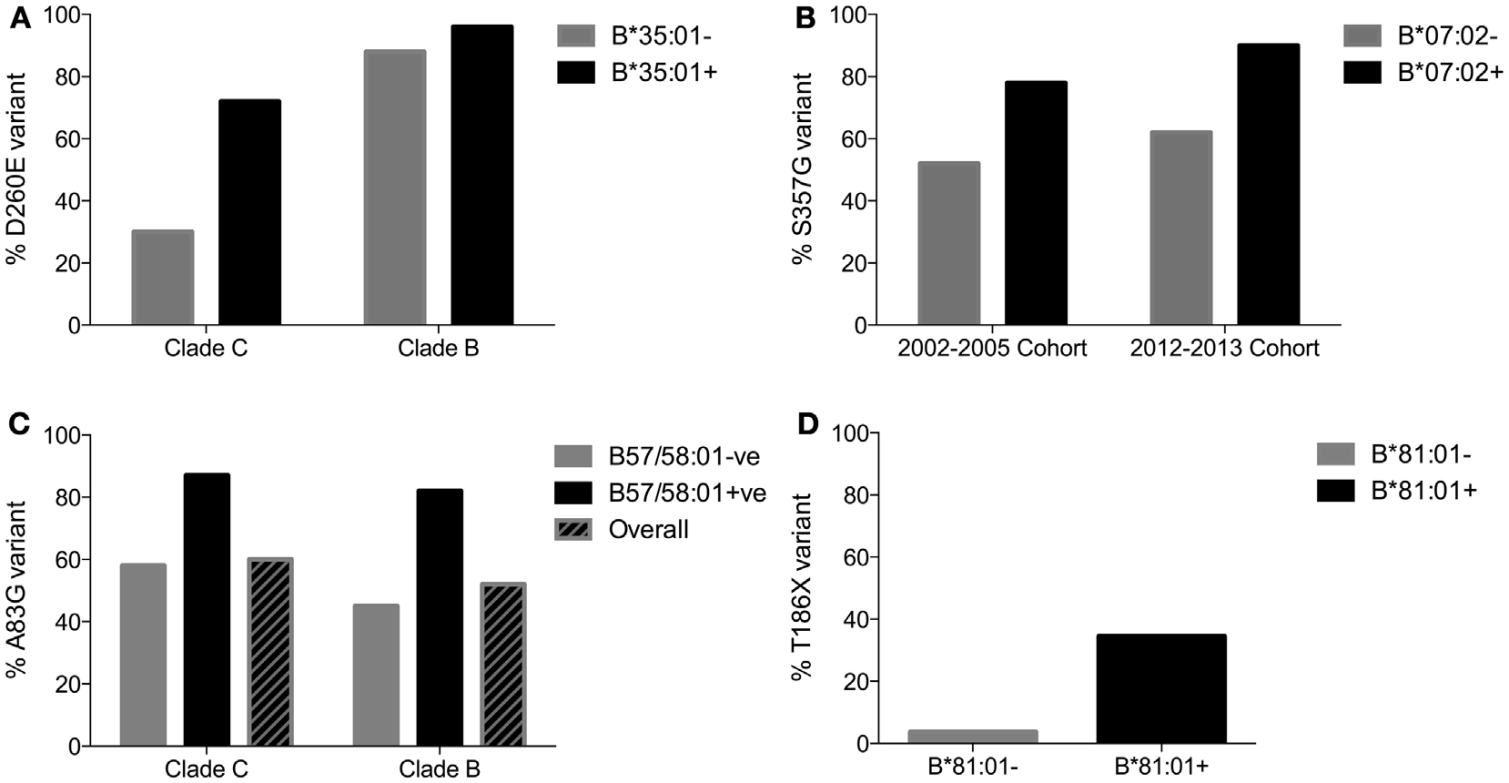
**Variable speed of variant accumulation**. **(A)** Frequency of HLA-B*35:01-driven D260E variant in a C clade-infected population in which HLA-B*35:01 is at low prevalence (phenotypic frequency 4%) compared to a B clade-infected population in which HLA-B*35:01 is at high prevalence (15%). **(B)** Frequency of HLA-B*07:02-driven S357G variant in two comparable Durban cohorts, enrolled in 2002–2005 and 2012–2013. These examples shown in **(A,B)** illustrate escape mutants selected early in the course of infection by a high proportion of subjects expressing the relevant HLA allele, which confer little fitness cost on the virus. Therefore, the variant accumulates rapidly in the population. **(C)** Frequency of HLA-B*57/58:01 A83G variant in two cohorts. The third example of rapid accumulation of an escape variant selected early in the course of infection, conferring little fitness cost that accumulates rapidly in the population. **(D)** Frequency of HLA-B*81:01-driven variant T186X. This example illustrates a variant conferring a high fitness cost on the virus, but for which compensatory mutations have only very modest effects. Therefore, the T186X variant accumulates very slowly in the population. Data from Kawashima et al. (47), Leslie et al. (93), and Payne et al. (96).

Other factors than the simple accumulation of escape mutants over time might contribute to the presence of an escape mutant at high frequencies in the population include, in particular, founder effect. For this reason, it is necessary for sequences to be “phylogenetically corrected” (94) in order to exclude founder effect and verify identified HLA associations. An example here would be the presence of a handful of C clade sequences from infected Africans among a large cohort of mostly B-clade-infected Caucasians. Without phylogenetic correction, the analysis would generate artifactual HLA associations between HLA alleles prevalent in Africans (such as HLA-C*17:01) and polymorphisms specific to C clade virus, when no biological link exists. The persistence of HLA associations following phylogenetic correction supports the notion that HLA, if not the only driving force, is at least contributing to the accumulation of escape mutants in the population. An example here may be the HLA-B*35:01 escape mutant D260E within the NY10 epitope (NPPIPVGDIY, Gag 254–262; Figure 3). In C clade infection, while 72% of HLA-B*35:01-positive individuals carry the escape mutant D260E, Gag-260-D is present in 70% of sequences and is therefore the consensus. However, in B clade infection, 96% of HLA-B*35:01-positive individuals carry D260E, Gag-260-D is present in only 12% of sequences, and Gag-260-E is therefore the consensus. Since this D260E mutant appears to carry no fitness cost (68), it is likely that HLA-B*35:01-NY10-specific CD8^+^ T-cell activity is contributing to the accumulation of D260E in B and C clade virus populations (95).

The second line of evidence suggesting that CD8^+^ T-cell escape mutants may be accumulating at the population level over the course of the epidemic derives from the observation, among nine ethnically diverse cohorts of HIV-infected individuals, of a strong correlation between the frequency of escape mutants and the prevalence of the relevant HLA molecule in each study cohort (47). In Japan, for example, where HLA-B*51 is expressed in 20–25% of the population, the HLA-B*51-associated escape mutant Pol-I135X is present in two-thirds of HLA-B*51-negative population. I135X is selected in acute infection in >95% of HIV-infected, HLA-B*51-positive individuals, and the I135X variants, with one rare exception, have no impact on viral fitness. Hence, the conditions for rapid accumulation of this variant at the population level are met (97) (Figure 3). In Caucasian populations where HLA-B*51 prevalence is ~10%, the frequency of I135X is ~30% in HLA-B*51-negative individuals; and in African populations where the HLA-B*51 phenotypic frequency is low (1–4%), the frequency of the I135X variant is also correspondingly low (~15%). Surprisingly, these studies additionally showed evidence of accumulation of escape mutants known to carry a fitness cost on the virus, although as expected these appeared to accumulate at slower rates than the mutants with no fitness cost attached.

The third study, comparing the frequency of escape mutants at the population level at the same location in Durban, South Africa, at two time points 10 years apart (2002–2005 and 2012–2013), showed a significantly increased frequency of CD8^+^ T-cell escape mutants, further supporting the notion that these escape mutants are increasing at the population level over time (96). A similar study of the North American epidemic (98) also showed an increase in the frequency of escape mutants over time, although the relatively modest twofold increase in frequency over a 20-year period may reflect a less dramatic HIV epidemic than that experienced in sub-Saharan Africa. In addition, the ethnically homogeneous Zulu cohorts in Durban, relative to the ethnically heterogeneous cohorts in North America, might generate a more focused selection pressure and therefore a speedier accumulation of mutants in the African population.

Thus, several lines of evidence point to an accumulation of escape mutants over time in different cohorts, although rate of accumulation might vary according to the population structure, the scale of the epidemic, and the nature of the particular escape mutant (Figure 3).

### Population-Level Accumulation of Escape Mutants and Loss of Protective HLA Impact

One might predict, from the data presented above, that escape mutants would accumulate over the course of the HIV epidemic, albeit at differing rates, and that this would have two potential consequences. First, if the HLA associations with slow disease progression, such as have been described for HLA-B*27, hinge to any extent on particular epitopes, the loss of those epitopes might be likely to affect the HLA association with disease outcome. Second, if the escape mutants that accumulate at the population level carry a fitness cost compared to the original “wild-type” virus, this would tend to bring the average replicative capacity of the circulating viruses in the population down. Evidence for these two possibilities will be discussed below.

The clearest evidence that HLA associations with HIV disease outcome are influenced by the availability of certain CTL epitopes comes from the SIV-macaque model of HIV infection. The MHC molecule Mamu-B*08 in SIV-infected Indian rhesus macaques is similar in several respects to HLA-B*27 in HIV-infected humans. First, it is associated with successful suppression of viremia and with slow progression in SIV infection (99). Second, the Mamu-B*08 peptide-binding motif is so similar to HLA-B*27 that in some cases, the same peptides can bind both Mamu-B*08 and HLA-B*27; in fact, the SIV-specific Mamu-B*08-restricted CD8^+^ T-cell epitopes include the exact homologs of the HIV-specific HLA-B*27-restricted epitopes (100). Finally, the dominant Mamu-B*08-restricted CD8^+^ T-cell epitope is located in a highly conserved region of the proteome, escape from which is therefore limited by fitness constraints. In the SIV model, the question could then be addressed of whether control of SIV infection would be lost if viruses carrying escape mutants in all the critical Mamu-B*08 epitopes were engineered into the infecting strain of virus. This experiment was performed (101) and the result was that control of SIV was indeed compromised by loss of these epitopes. Furthermore, failure to control this virus was in spite of the reduced VRC incurred as a consequence of all the additional mutants. Although the experiment was not done, it is possible that this “escaped” virus might have been well contained in Mamu-B*08-negative animals, owing to the lower VRC.

In HIV infection, examples of the impact of transmission of CTL escape mutants to HLA-matched individuals initially were mostly anecdotal. The negation of the protective effect of HLA-B*27 in children whose mothers transmit the KK10 escape variants has already been described above (79). Larger studies of mother–child pairs support these initial findings (80). In adults, studies of epidemiologically linked Zambian transmission pairs also indicate that disease progression is more rapid following transmission of CTL escape variants to HLA-matched recipients than following transmission of the wild-type virus (43, 82; Carlson, Personal communication). Thus, the evidence does suggest that immune control is reduced and HLA associations with slow progression would be disrupted by the accumulation of escape mutations over the course of the epidemic.

The second line of evidence supporting the data from transmission pairs is that emerging from studies of clade-specific differences in HLA associations with particular HIV disease outcomes. An example has been mentioned of the difference between B and C clade HIV with respect to the residue at Gag-260: in B clade, this is Glu in 88% of cases, whereas in C clade, this residue is Asp in 70%. The Gag epitope NPPIPVGDIY (“NY10”; Gag 254–262) is only immunogenic when Asp is present at Gag-260, and hence the great majority of B clade-infected individuals who are HLA-B*35:01-positive cannot make a response to NY10. However, in C clade infection, the majority if HLA-B*35:01-positive individuals can make a response to NY10. Since this NY10 response is associated with a 1log^10^ reduction in viral load (95), it is likely that this contributes to the differential clade-specific HLA-B*35:01 association with HIV disease outcome, that is, with rapid progression in B clade infection and no association with rapid progression in C clade infection. Similar data are published with respect to the HLA-B*07:02-restricted p24 Gag-specific epitope, which is immunogenic in C clade infection but “escaped” and non-immunogenic in B clade infection (102). In parallel, the association of HLA-B*07:02 with rapid progression in B clade infection is not observed in C clade infection. Therefore, these data support the notion that infection with a virus that is preadapted to the HLA type of the recipient will typically have two results. First, infection will result in worse outcome. Second, the prevailing HLA associations with protection against disease progression will be disrupted.

The data that HLA associations with HIV disease outcome could already be altering over time are, as one might expect, somewhat limited after the relatively short timescales over which the HIV epidemic has been operating. However, there is evidence that in the 1980s in Japan, early in the epidemic, HLA-B*51 was associated with slow progression in HIV infection (12, 14, 47), whereas 25 years later, it no longer affords any protective effect (47). This loss of protection mediated through HLA-B*51 over a 25-year period in Japan is associated with the apparent rise in frequency of the I135X mutation abrogating binding of the dominant TAFTIPSI (RT 128–135) epitope described above. Consistent with this picture are the data indicating that non-progression in subjects expressing HLA-B*51 may hinge on the lack of escape in the TAFTIPSI epitope (103).

More recently, Payne et al. (96) studied the impact of HIV on HLA adaptation in Botswana, one of the countries most severely affected by the epidemic, with high seroprevalence taking off in the 1980s and rates of adult infection that approached 30% by 2000. Even today, adult seroprevalence in Botswana remains at 23%. Although South Africa is the country with the highest absolute number of HIV infections, exceeding >6 m people currently,^1^ the duration of the epidemic and the magnitude in terms of adult seroprevalence is substantially less than that in Botswana (WHO Report 2013^2^). As expected, the degree to which HIV at the population level is adapted to the HLA class I molecules expressed in the infected individuals in Botswana was substantially higher than that in South Africa. Of note, this increased population-level adaptation of HLA also applied to the related protective HLA alleles HLA-B*57 and HLA-B*58:01, although not to the protective HLA-B*81:01. This finding prompted the question of whether the protection against disease progression that is typically mediated by HLA-B*57/58:01 is diminished in Botswana compared to South Africa. Strikingly, there was no significant protection against disease progression, as indicated by viral load, afforded by HLA-B*57/58:01 in Botswana. However, in the case of HLA-B*81:01, this allele remained protective in Botswana as in South Africa.

This is a striking result because HLA-B*57/58:01 has been universally associated with the successful suppression of viremia in all the previous studies where sufficient subject numbers have been sampled (12, 15, 16, 35–40). The number of subjects studied in Botswana was relatively high (*n* > 400), of whom 90 expressed HLA-B*57/58:01. As described above, there is evidence also from comparable cohorts in Durban recruited, respectively, in 2002–2005 and in 2012–2013, that CD8^+^ T-cell escape mutants are accumulating, and one would predict therefore that loss of HLA-B*57/58:01 protection against disease progression would eventually be demonstrated also in South Africa.

It might initially appear surprising that HIV would be able to adapt to certain HLA alleles, such as HLA-B*57/58:01, and not others, such as HLA-B*81. However, as described above (see Figure 3), it is well documented that certain escape variants are selected at very high frequency, early in the course of infection, and incur little fitness cost to the virus, and hence would accumulate rapidly, whereas others would accumulate substantially more slowly. The HLA-B*81:01-driven escape mutant T186X within the dominant Gag epitope TL9 (TPQDLNTML, Gag 180–188) may be such an example (Figure 3), being present in only 25–30% of chronically infected subjects expressing HLA-B*81:01, and carrying a high fitness cost, without clear compensatory mutants being identified to date (60; Tsai, Personal communication). So, accumulation of this latter escape mutant would be slow, and HLA-B*81:01 may remain a long-term protective HLA allele in HIV infection as a result.

### Population-Level Accumulation of Escape Mutants and Impact on HIV Virulence

#### Markers of Virulence

The population-level adaptation of HIV to a high proportion of all the HIV-specific CD8^+^ T-cell responses generated against the virus, including most effective responses that are restricted by HLA-B*57/58:01, prompts the question of what impact do these events have on virulence of the epidemic. Virulence refers to the ability of a microorganism to cause disease (104). Both viral setpoint and CD4 count have been used as proxy measures of virulence, since the former predicts rate of progression (105) and the latter indicates risk of opportunistic infections (106); indeed, a CD4 count of <200 cells/mm^3^ is one definition of AIDS. However, it is worth noting that these measures are not synonymous with disease progression (107). Indeed, it has become increasingly apparent that immune activation is a better marker than viral load of disease outcome (108). The most obvious example of this is natural SIV infection in African non-human primates, such as the sooty mangabey and the African green monkey, in which non-pathogenic infection operates in the setting of persistent, high viremia and low immune activation (109, 110). Non-progressive pediatric HIV infection is an example in humans of the same disconnection between viral load and disease outcome, normal CD4 T-cell count and function, and low immune activation in the setting of persistent high viremia (Adland, Personal ­communication). In adult HIV infection, immune activation levels predict disease outcome better than viral load (108, 111), and CD4 decline to AIDS can be observed in elite controllers (whose viral load is undetectable) as a result of the strong correlation between absolute CD4 count and immune activation (112).

The underlying mechanism by which immune activation is maintained at low levels in the setting of high viremia in the sooty mangabey and African green monkey appears to be related to strong immunoregulatory activity that follows a robust innate immune response to SIV in acute infection (110, 113). It is likely that host-specific variation in immune activation following HIV infection may, in part, explain differential disease outcome in HIV infection, although the clearest host genetic impact on rate of progression arises from differences in HLA class I molecules expressed (114). However, an additional possibility has recently been proposed that VRC of the transmitted virus has an impact not only on viral setpoint and CD4 count in the recipient (82, 84) but also more directly on the level of immune activation (115). This relationship between VRC of the founder virus and early immune activation in the recipient is independent of viral load. In addition, as described also in natural SIV infection (116), low immune activation in this study was associated with small viral reservoirs in naïve and central memory CD4^+^ T-cells. This helps to explain why donor and recipient viral load in transmission pairs are strongly correlated (117). Therefore, together these studies would indicate that VRC may be a stronger predictor of virulence than viral load.

#### Changing Virulence in the HIV Epidemic – HLA Effects

The accumulation of escape mutants, including those reducing the replicative capacity of HIV, would suggest the possibility that population-level VRC would decline over time (118). The study of Payne et al. (96), referred to previously, showed that the substantially higher level of population-level adaptation of HIV observed in Botswana compared to South Africa was indeed associated with a significantly lower median VRC. As indicated above, VRC may be a more direct indicator than the other measurements used as a proxy for virulence, namely viral load and CD4 count (115), although correlations between VRC and viral load, and/or inverse correlations between VRC and CD4 count are described in all published studies to date (50, 58, 60, 96, 119–123), including that of Payne et al. (96). These data in Botswana suggested that time to AIDS in the absence of ART would be extended on average by ~2.5 years (124) as a result of the viral adaptation observed.

#### Changing Virulence in the HIV Epidemic – Non-HLA Effects

A further question here is whether the lowered VRC in Botswana was entirely due to the differential population-level HLA adaptation or whether other factors may have contributed. It seems likely that more widespread use of ART, as has occurred in Botswana compared to South Africa, would result in lowering of population-level VRC. In the absence of ART, the fittest viruses are those associated with high viral load and low CD4 count, and therefore these are the viruses most likely to be transmitted. In a population in whom ART is systematically given to infected subjects with high viral loads and low CD4 counts, the transmission rates overall will be reduced, but the viruses transmitted will tend to arise from donors who have relatively high CD4 counts and therefore relatively low fitness viruses. Mathematical modeling of the impact of ART in virulence also appears to support this hypothesis (96).

Several other studies have been undertaken that have examined the question of whether HIV virulence is changing over the course of the epidemic, and, if so, in what direction. Herbeck et al. (104) published a review of 32 European/North American studies on the B clade epidemic, 12 of which indicated that there was no change, nine indicated an increase, and 11 a decrease in virulence. Subsequent to this review, further studies have suggested a decrease in virulence in the HIV epidemic in Japan, as determined by chance population-level VRC (122), and the study of the mainly European CASCADE cohort [Concerted Action on SeroConversion to AIDS and Death in Europe (125)] indicated increasing virulence from the early 1980s to 2002, followed by a plateau or slight decline thereafter in CD4 counts and viral load, respectively.

These studies undertaking estimates of changing virulence face several challenges, including population heterogeneity, the discrepancies between different viral load testing platforms, accurate definition of seroconversion date, and taking account of the impact of widely diverse ART initiation strategies and guidelines. However, the CASCADE study adjusted for possible confounders using several approaches, and it is useful to consider other possible reasons why the findings of these various studies might be so diametrically opposite.

One major potential factor is clearly the very different type of epidemic operating in the European and North America versus the sub-Saharan Africa: the sub-Saharan epidemic is mainly comprised of women infected with C clade virus via sex with men, whereas the majority of study subjects in European/North American studies are of B clade-infected men who have sex with men (MSM). These differences alone will bring a number of other factors into the equation, such as the transmission of other viral infections, such as CMV, HSV2, hepatitis B, and hepatitis C, all of which have a detrimental impact on the ability of the immune response to control HIV infection (126). In these instances, measurement of VRC might indeed provide a closer estimate of virulence. In the case of CMV, in sub-Saharan Africa, virtually all individuals are infected by the age of 12 months, whereas in Europe and North America, only 50% of adults are CMV seropositive. The impact of comorbidity is difficult to evaluate; however, since poverty, malnutrition, and preexisting high prevalence of diseases, such as TB and hepatitis B, in sub-Saharan Africa would tend to decrease mucosal barriers to infection, and potentially enable lower fitness viruses to be transmitted that would not be transmitted in the setting of a healthy mucosal barrier.

The impact of wide availability of antiretroviral drugs for the past 20 years for infected individuals in Europe and North America, and not to the same extent in sub-Saharan Africa, is likely to increase the proportion of transmissions that occur in acute or early infection, and therefore involving the fittest and most virulent viruses. The estimates of the percentage of transmissions that occur during acute infection in the donor range widely between studies, from 1 to 50%, with a consensus figure of ~25% (127–134). If the proportion of transmissions occurring during acute infection is higher in the MSM epidemic, as appears likely, especially in the European/North American epidemic where later infection is blocked by a very efficient treatment program, this might tend to favor increasing virulence. Furthermore, in relation to the rate of accumulating CTL escape mutants, early transmission would likely preempt the serial passaging of virus from one individual to another before the action of the cellular immune response on the virus, also diminishing the HLA impact on virulence described above.

## Conclusion

HLA has had a dramatic impact in a short period of time in shaping evolution of HIV as it has adapted to evade the most effective CD8^+^ T-cell responses generated against it. In Southern Africa, where the epidemic has most severely affected populations, there is evidence that accumulating escape mutants in some of the critical epitopes for immune control is associated with diminished protection afforded by the restricting HLA alleles. However, although HLA alleles, such as HLA-B*57, may be losing its protective impact, nonetheless, the legacy to the population of the accumulating HLA-B*57-driven mutants is of a gradual lowering of the population-level viral fitness. As ART coverage increases dramatically in these parts of the world where the epidemic is most highly concentrated, so it is likely that the proportion of transmissions that arise in acute infection may increase. If this is the case, it is possible that HLA may have less of a role than hitherto. However, the goal of reaching all HIV-infected individuals with ART, and maintaining viral suppression in those treated individuals, is an extraordinary challenge, and it remains to be seen what impact HLA will have on HIV evolution in the future.

## Author Contributions

HK contributed to the manuscript writing. AL contributed with ideas, editorial inputs, and critical reviewing of the draft. PG wrote the majority of the manuscript and outlined the review topic.

## Conflict of Interest Statement

The authors declare that the research was conducted in the absence of any commercial or financial relationships that could be construed as a potential conflict of interest.

## Footnotes

^1^www.avert.org

^2^www.who.int/hiv

## References

[1] Buchbinder SP Mehrotra DV Duerr A Fitzgerald DW Mogg R Li D Efficacy assessment of a cell-mediated immunity HIV-1 vaccine (the step study): a double-blind, randomised, placebo-controlled, test-of-concept trial. Lancet (2008) 372:1881–93. 10.1016/S0140-6736(08)61591-3 19012954 PMC2721012

[2] Gray G Buchbinder S Duerr A. Overview of STEP and Phambili trial results: two phase IIb test-of-concept studies investigating the efficacy of MRK adenovirus type 5 gag/pol/nef subtype B HIV vaccine. Curr Opin HIV AIDS (2010) 5:357–61. 10.1097/COH.0b013e32833d2d2b 20978374 PMC2995949

[3] Gray GE Allen M Moodie Z Churchyard G Bekker LG Nchabeleng M Safety and efficacy of the HVTN 503/Phambili study of a clade-B-based HIV-1 vaccine in South Africa: a double-blind, randomised, placebo-­controlled test-of-concept phase 2b study. Lancet Infect Dis (2011) 11:507–15. 10.1016/S1473-3099(11)70098-6 21570355 PMC3417349

[4] Gray GE Moodie Z Metch B Gilbert PB Bekker LG Churchyard G Recombinant adenovirus type 5 HIV gag/pol/nef vaccine in South Africa: unblinded, long-term follow-up of the phase 2b HVTN 503/Phambili study. Lancet Infect Dis (2014) 14:388–96. 10.1016/S1473-3099(14)70020-9 24560541 PMC4174314

[5] Hansen SG Vieville C Whizin N Coyne-Johnson L Siess DC Drummond DD Effector memory T cell responses are associated with protection of rhesus monkeys from mucosal simian immunodeficiency virus challenge. Nat Med (2009) 15:293–9. 10.1038/nm.1935 19219024 PMC2720091

[6] Hansen SG Ford JC Lewis MS Ventura AB Hughes CM Coyne-Johnson L Profound early control of highly pathogenic SIV by an effector memory T-cell vaccine. Nature (2011) 473:523–7. 10.1038/nature10003 21562493 PMC3102768

[7] Hansen SG Piatak M Jr Ventura AB Hughes CM Gilbride RM Ford JC Immune clearance of highly pathogenic SIV infection. Nature (2013) 502:100–4. 10.1038/nature12519 24025770 PMC3849456

[8] Hansen SG Sacha JB Hughes CM Ford JC Burwitz BJ Scholz I Cytomegalovirus vectors violate CD8+ T cell epitope recognition paradigms. Science (2013) 340:1237874 10.1126/science.1237874 23704576 PMC3816976

[9] Deeks SG HIV: shock and kill. Nature (2012) 487:439–40. 10.1038/487439a 22836995

[10] Deng K Pertea M Rongvaux A Wang L Durand CM Ghiaur G Broad CTL response is required to clear latent HIV-1 due to dominance of escape mutations. Nature (2015) 517:381–5. 10.1038/nature14053 25561180 PMC4406054

[11] Phillips RE Rowland-Jones S Nixon DF Gotch FM Edwards JP Ogunlesi AO Human immunodeficiency virus genetic variation that can escape cytotoxic T cell recognition. Nature (1991) 354:453–9. 10.1038/354453a0 1721107

[12] Kaslow RA Carrington M Apple R Park L Munoz A Saah AJ Influence of combinations of human major histocompatibility complex genes on the course of HIV-1 infection. Nat Med (1996) 2:405–11. 10.1038/nm0496-405 8597949

[13] Goulder PJ Phillips RE Colbert RA Mcadam S Ogg G Nowak MA Late escape from an immunodominant cytotoxic T-lymphocyte response associated with progression to AIDS. Nat Med (1997) 3:212–7. 10.1038/nm0297-212 9018241

[14] O’Brien SJ Gao X Carrington M. HLA and AIDS: a cautionary tale. Trends Mol Med (2001) 7:379–81. 10.1016/S1471-4914(01)02131-1 11530315

[15] Fellay J Shianna KV Ge D Colombo S Ledergerber B Weale M A whole-genome association study of major determinants for host control of HIV-1. Science (2007) 317:944–7. 10.1126/science.1143767 17641165 PMC1991296

[16] Pereyra F Jia X Mclaren PJ Telenti A De Bakker PI Walker BD The major genetic determinants of HIV-1 control affect HLA class I peptide presentation. Science (2010) 330:1551–7. 10.1126/science.1195271 21051598 PMC3235490

[17] Jardetzky TS Lane WS Robinson RA Madden DR Wiley DC. Identification of self peptides bound to purified HLA-B27. Nature (1991) 353:326–9. 10.1038/353326a0 1922338

[18] Madden DR Gorga JC Strominger JL Wiley DC. The three-­dimensional structure of HLA-B27 at 2.1 A resolution suggests a general mechanism for tight peptide binding to MHC. Cell (1992) 70:1035–48. 10.1016/0092-8674(92)90252-8 1525820

[19] Rotzschke O Falk K Stevanovic S Gnau V Jung G Rammensee HG Dominant aromatic/aliphatic C-terminal anchor in HLA-B*2702 and B*2705 peptide motifs. Immunogenetics (1994) 39:74–7. 10.1007/BF00171803 8225441

[20] Marsh SGE Parham P Barber LD The HLA Facts Book. London: Academic Press (2000).

[21] Nixon DF Townsend AR Elvin JG Rizza CR Gallwey J Mcmichael AJ. HIV-1 gag-specific cytotoxic T lymphocytes defined with recombinant vaccinia virus and synthetic peptides. Nature (1988) 336:484–7. 10.1038/336484a0 2461519

[22] Payne RP Kloverpris H Sacha JB Brumme Z Brumme C Buus S Efficacious early antiviral activity of HIV Gag- and Pol-specific HLA-B*2705-restricted CD8+ T cells. J Virol (2010) 84:10543–57. 10.1128/JVI.00793-10 20686036 PMC2950555

[23] Kelleher AD Long C Holmes EC Allen RL Wilson J Conlon C Clustered mutations in HIV-1 gag are consistently required for escape from HLA-B27-restricted cytotoxic T lymphocyte responses. J Exp Med (2001) 193:375–86. 10.1084/jem.193.3.375 11157057 PMC2195921

[24] O’Brien KL Liu J King SL Sun YH Schmitz JE Lifton MA Adenovirus-specific immunity after immunization with an Ad5 HIV-1 vaccine candidate in humans. Nat Med (2009) 15:873–5. 10.1038/nm.1991 19620961 PMC2756115

[25] Appay V Nixon DF Donahoe SM Gillespie GM Dong T King A HIV-specific CD8(+) T cells produce antiviral cytokines but are impaired in cytolytic function. J Exp Med (2000) 192:63–75. 10.1084/jem.192.1.63 10880527 PMC1887711

[26] Almeida JR Price DA Papagno L Arkoub ZA Sauce D Bornstein E Superior control of HIV-1 replication by CD8+ T cells is reflected by their avidity, polyfunctionality, and clonal turnover. J Exp Med (2007) 204:2473–85. 10.1084/jem.20070784 17893201 PMC2118466

[27] Almeida JR Sauce D Price DA Papagno L Shin SY Moris A Antigen sensitivity is a major determinant of CD8+ T-cell polyfunctionality and HIV-suppressive activity. Blood (2009) 113:6351–60. 10.1182/blood-2009-02-206557 19389882 PMC2710928

[28] Berger CT Frahm N Price DA Mothe B Ghebremichael M Hartman KL High-functional-avidity cytotoxic T lymphocyte responses to HLA-B-restricted Gag-derived epitopes associated with relative HIV control. J Virol (2011) 85:9334–45. 10.1128/JVI.00460-11 21752903 PMC3165743

[29] Iglesias MC Almeida JR Fastenackels S Van Bockel DJ Hashimoto M Venturi V Escape from highly effective public CD8+ T-cell clonotypes by HIV. Blood (2011) 118:2138–49. 10.1182/blood-2011-01-328781 21734237 PMC3162351

[30] Ladell K Hashimoto M Iglesias MC Wilmann PG Mclaren JE Gras S A molecular basis for the control of preimmune escape variants by HIV-specific CD8+ T cells. Immunity (2013) 38:425–36. 10.1016/j.immuni.2012.11.021 23521884

[31] Schneidewind A Brockman MA Yang R Adam RI Li B Le Gall S Escape from the dominant HLA-B27-restricted cytotoxic T-lymphocyte response in Gag is associated with a dramatic reduction in human immunodeficiency virus type 1 replication. J Virol (2007) 81:12382–93. 10.1128/JVI.01543-07 17804494 PMC2169010

[32] Schneidewind A Brockman MA Sidney J Wang YE Chen H Suscovich TJ Structural and functional constraints limit options for cytotoxic T-lymphocyte escape in the immunodominant HLA-B27-restricted epitope in human immunodeficiency virus type 1 capsid. J Virol (2008) 82:5594–605. 10.1128/JVI.02356-07 18385228 PMC2395179

[33] Chen H Ndhlovu ZM Liu D Porter LC Fang JW Darko S TCR clonotypes modulate the protective effect of HLA class I molecules in HIV-1 infection. Nat Immunol (2012) 13:691–700. 10.1038/ni.2342 22683743 PMC3538851

[34] Shasha D Walker BD Lessons to be learned from natural control of HIV – future directions, therapeutic, and preventive implications. Front Immunol (2013) 4:162 10.3389/fimmu.2013.00162 23805139 PMC3691556

[35] Goulder PJ Bunce M Krausa P Mcintyre K Crowley S Morgan B Novel, cross-restricted, conserved, and immunodominant cytotoxic T lymphocyte epitopes in slow progressors in HIV type 1 infection. AIDS Res Hum Retroviruses (1996) 12:1691–8. 10.1089/aid.1996.12.1691 8959245

[36] Migueles SA Sabbaghian MS Shupert WL Bettinotti MP Marincola FM Martino L HLA B*5701 is highly associated with restriction of virus replication in a subgroup of HIV-infected long term nonprogressors. Proc Natl Acad Sci U S A (2000) 97:2709–14. 10.1073/pnas.050567397 10694578 PMC15994

[37] Carrington M O’Brien SJ. The influence of HLA genotype on AIDS. Annu Rev Med (2003) 54:535–51. 10.1146/annurev.med.54.101601.152346 12525683

[38] Kiepiela P Leslie AJ Honeyborne I Ramduth D Thobakgale C Chetty S Dominant influence of HLA-B in mediating the potential co-evolution of HIV and HLA. Nature (2004) 432:769–75. 10.1038/nature03113 15592417

[39] Fellay J Ge D Shianna KV Colombo S Ledergerber B Cirulli ET Common genetic variation and the control of HIV-1 in humans. PLoS Genet (2009) 5:e1000791. 10.1371/journal.pgen.1000791 20041166 PMC2791220

[40] Thomas R Apps R Qi Y Gao X Male V O’Huigin C HLA-C cell surface expression and control of HIV/AIDS correlate with a variant upstream of HLA-C. Nat Genet (2009) 41:1290–4. 10.1038/ng.486 19935663 PMC2887091

[41] Kloverpris HN Stryhn A Harndahl M Van Der Stok M Payne RP Matthews PC HLA-B*57 micropolymorphism shapes HLA allele-specific epitope immunogenicity, selection pressure, and HIV immune control. J Virol (2012) 86:919–29. 10.1128/JVI.06150-11 22090105 PMC3255844

[42] Leslie AJ Pfafferott KJ Chetty P Draenert R Addo MM Feeney M HIV evolution: CTL escape mutation and reversion after transmission. Nat Med (2004) 10:282–9. 10.1038/nm992 14770175

[43] Crawford H Lumm W Leslie A Schaefer M Boeras D Prado JG Evolution of HLA-B*5703 HIV-1 escape mutations in HLA-B*5703-positive individuals and their transmission recipients. J Exp Med (2009) 206:909–21. 10.1084/jem.20081984 19307327 PMC2715113

[44] Carlson JM Listgarten J Pfeifer N Tan V Kadie C Walker BD Widespread impact of HLA restriction on immune control and escape pathways of HIV-1. J Virol (2012) 86:5230–43. 10.1128/JVI.06728-11 22379086 PMC3347390

[45] Martinez-Picado J Prado JG Fry EE Pfafferott K Leslie A Chetty S Fitness cost of escape mutations in p24 Gag in association with control of human immunodeficiency virus type 1. J Virol (2006) 80:3617–23. 10.1128/JVI.80.7.3617-3623.2006 16537629 PMC1440414

[46] Brackenridge S Evans EJ Toebes M Goonetilleke N Liu MK Di Gleria K An early HIV mutation within an HLA-B*57-restricted T cell epitope abrogates binding to the killer inhibitory receptor 3DL1. J Virol (2011) 85:5415–22. 10.1128/JVI.00238-11 21430058 PMC3094954

[47] Kawashima Y Pfafferott K Frater J Matthews P Payne R Addo M Adaptation of HIV-1 to human leukocyte antigen class I. Nature (2009) 458:641–5. 10.1038/nature07746 19242411 PMC3148020

[48] Boutwell CL Rowley CF Essex M. Reduced viral replication capacity of human immunodeficiency virus type 1 subtype C caused by cytotoxic-T-­lymphocyte escape mutations in HLA-B57 epitopes of capsid protein. J Virol (2009) 83:2460–8. 10.1128/JVI.01970-08 19109381 PMC2648284

[49] Boutwell CL Carlson JM Lin TH Seese A Power KA Peng J Frequent and variable cytotoxic-T-lymphocyte escape-associated fitness costs in the human immunodeficiency virus type 1 subtype B Gag proteins. J Virol (2013) 87:3952–65. 10.1128/JVI.03233-12 23365420 PMC3624202

[50] Miura T Brumme CJ Brockman MA Brumme ZL Pereyra F Block BL HLA-associated viral mutations are common in human immunodeficiency virus type 1 elite controllers. J Virol (2009) 83:3407–12. 10.1128/JVI.02459-08 19153230 PMC2655568

[51] Troyer RM Mcnevin J Liu Y Zhang SC Krizan RW Abraha A Variable fitness impact of HIV-1 escape mutations to cytotoxic T lymphocyte (CTL) response. PLoS Pathog (2009) 5:e1000365. 10.1371/journal.ppat.1000365 19343217 PMC2659432

[52] Kiepiela P Ngumbela K Thobakgale C Ramduth D Honeyborne I Moodley E CD8+ T-cell responses to different HIV proteins have discordant associations with viral load. Nat Med (2007) 13:46–53. 10.1038/nm1520 17173051

[53] Santiago ML Rodenburg CM Kamenya S Bibollet-Ruche F Gao F Bailes E SIVcpz in wild chimpanzees. Science (2002) 295:465 10.1126/science.295.5554.465 11799233

[54] Wroblewski EE Norman PJ Guethlein LA Rudicell RS Ramirez MA Li Y Signature patterns of MHC diversity in three Gombe communities of wild chimpanzees reflect fitness in reproduction and immune defense against SIVcpz. PLoS Biol (2015) 13:e1002144. 10.1371/journal.pbio.1002144 26020813 PMC4447270

[55] Leslie A Matthews PC Listgarten J Carlson JM Kadie C Ndung’u T Additive contribution of HLA class I alleles in the immune control of HIV-1 infection. J Virol (2010) 84:9879–88. 10.1128/JVI.00320-10 20660184 PMC2937780

[56] Prentice HA Porter TR Price MA Cormier E He D Farmer PK HLA-B*57 versus HLA-B*81 in HIV-1 infection: slow and steady wins the race? J Virol (2013) 87:4043–51. 10.1128/JVI.03302-12 23365442 PMC3624227

[57] Goulder PJ Brander C Annamalai K Mngqundaniso N Govender U Tang Y Differential narrow focusing of immunodominant human immunodeficiency virus gag-specific cytotoxic T-lymphocyte responses in infected African and Caucasoid adults and children. J Virol (2000) 74:5679–90. 10.1128/JVI.74.12.5679-5690.2000 10823876 PMC112056

[58] Huang KH Goedhals D Carlson JM Brockman MA Mishra S Brumme ZL Progression to AIDS in South Africa is associated with both reverting and compensatory viral mutations. PLoS One (2011) 6:e19018. 10.1371/journal.pone.0019018 21544209 PMC3081339

[59] Kloverpris HN Cole DK Fuller A Carlson J Beck K Schauenburg AJ A molecular switch in immunodominant HIV-1-specific CD8 T-cell epitopes shapes differential HLA-restricted escape. Retrovirology (2015) 12:20 10.1186/s12977-015-0149-5 25808313 PMC4347545

[60] Wright JK Naidoo VL Brumme ZL Prince JL Claiborne DT Goulder PJ Impact of HLA-B*81-associated mutations in HIV-1 Gag on viral replication capacity. J Virol (2012) 86:3193–9. 10.1128/JVI.06682-11 22238317 PMC3302318

[61] Kloverpris HN Mcgregor R Mclaren JE Ladell K Harndahl M Stryhn A CD8+ TCR bias and immunodominance in HIV-1 infection. J Immunol (2015) 194:5329–45. 10.4049/jimmunol.1400854 25911754 PMC4433859

[62] Leslie A Price DA Mkhize P Bishop K Rathod A Day C Differential selection pressure exerted on HIV by CTL targeting identical epitopes but restricted by distinct HLA alleles from the same HLA supertype. J Immunol (2006) 177:4699–708. 10.4049/jimmunol.177.12.8878-a 16982909

[63] Geldmacher C Metzler IS Tovanabutra S Asher TE Gostick E Ambrozak DR Minor viral and host genetic polymorphisms can dramatically impact the biologic outcome of an epitope-specific CD8 T-cell response. Blood (2009) 114:1553–62. 10.1182/blood-2009-02-206193 19542300 PMC2731637

[64] Kloverpris HN Harndahl M Leslie AJ Carlson JM Ismail N Van Der Stok M HIV control through a single nucleotide on the HLA-B locus. J Virol (2012) 86:11493–500. 10.1128/JVI.01020-12 22896606 PMC3486337

[65] Martin MP Gao X Lee JH Nelson GW Detels R Goedert JJ Epistatic interaction between KIR3DS1 and HLA-B delays the progression to AIDS. Nat Genet (2002) 31:429–34. 10.1038/ng934 12134147

[66] Gao X Nelson GW Karacki P Martin MP Phair J Kaslow R Effect of a single amino acid change in MHC class I molecules on the rate of progression to AIDS. N Engl J Med (2001) 344:1668–75. 10.1056/NEJM200105313442203 11386265

[67] Jin X Gao X Ramanathan M Jr Deschenes GR Nelson GW O’Brien SJ Human immunodeficiency virus type 1 (HIV-1)-specific CD8+-T-cell responses for groups of HIV-1-infected individuals with different HLA-B*35 genotypes. J Virol (2002) 76:12603–10. 10.1128/JVI.76.24.12603-12610.2002 12438586 PMC136673

[68] Matthews PC Prendergast A Leslie A Crawford H Payne R Rousseau C Central role of reverting mutations in HLA associations with human immunodeficiency virus set point. J Virol (2008) 82:8548–59. 10.1128/JVI.00580-08 18596105 PMC2519667

[69] Ngumbela KC Day CL Mncube Z Nair K Ramduth D Thobakgale C Targeting of a CD8 T cell env epitope presented by HLA-B*5802 is associated with markers of HIV disease progression and lack of selection pressure. AIDS Res Hum Retroviruses (2008) 24:72–82. 10.1089/aid.2007.0124 18275350

[70] Pereyra F Heckerman D Carlson JM Kadie C Soghoian DZ Karel D HIV control is mediated in part by CD8+ T-cell targeting of specific epitopes. J Virol (2014) 88:12937–48. 10.1128/JVI.01004-14 25165115 PMC4249072

[71] Apps R Meng Z Del Prete GQ Lifson JD Zhou M Carrington M. Relative expression levels of the HLA class-I proteins in normal and HIV-infected cells. J Immunol (2015) 194:3594–600. 10.4049/jimmunol.1403234 25754738 PMC4390493

[72] Apps R Qi Y Carlson JM Chen H Gao X Thomas R Influence of HLA-C expression level on HIV control. Science (2013) 340:87–91. 10.1126/science.1232685 23559252 PMC3784322

[73] Kulkarni S Savan R Qi Y Gao X Yuki Y Bass SE Differential microRNA regulation of HLA-C expression and its association with HIV control. Nature (2011) 472:495–8. 10.1038/nature09914 21499264 PMC3084326

[74] Kulkarni S Qi Y O’Huigin C Pereyra F Ramsuran V Mclaren P Genetic interplay between HLA-C and MIR148A in HIV control and Crohn disease. Proc Natl Acad Sci U S A (2013) 110:20705–10. 10.1073/pnas.1312237110 24248364 PMC3870724

[75] Carlson JM Brumme CJ Martin E Listgarten J Brockman MA Le AQ Correlates of protective cellular immunity revealed by analysis of population-level immune escape pathways in HIV-1. J Virol (2012) 86:13202–16. 10.1128/JVI.01998-12 23055555 PMC3503140

[76] Carlson JM Schaefer M Monaco DC Batorsky R Claiborne DT Prince J HIV transmission. Selection bias at the heterosexual HIV-1 transmission bottleneck. Science (2014) 345:1254031. 10.1126/science.1254031 25013080 PMC4289910

[77] Moore CB John M James IR Christiansen FT Witt CS Mallal SA. Evidence of HIV-1 adaptation to HLA-restricted immune responses at a population level. Science (2002) 296:1439–43. 10.1126/science.1069660 12029127

[78] Alter G Heckerman D Schneidewind A Fadda L Kadie CM Carlson JM HIV-1 adaptation to NK-cell-mediated immune pressure. Nature (2011) 476:96–100. 10.1038/nature10237 21814282 PMC3194000

[79] Goulder PJ Brander C Tang Y Tremblay C Colbert RA Addo MM Evolution and transmission of stable CTL escape mutations in HIV infection. Nature (2001) 412:334–8. 10.1038/35085576 11460164

[80] Thobakgale CF Prendergast A Crawford H Mkhwanazi N Ramduth D Reddy S Impact of HLA in mother and child on disease progression of pediatric human immunodeficiency virus type 1 infection. J Virol (2009) 83:10234–44. 10.1128/JVI.00921-09 19605475 PMC2748050

[81] Adland E Paioni P Thobakgale C Laker L Mori L Muenchhoff M Discordant impact of HLA on viral replicative capacity and disease progression in pediatric and adult HIV infection. PLoS Pathog (2015) 11:e1004954. 10.1371/journal.ppat.1004954 26076345 PMC4468173

[82] Goepfert PA Lumm W Farmer P Matthews P Prendergast A Carlson JM Transmission of HIV-1 Gag immune escape mutations is associated with reduced viral load in linked recipients. J Exp Med (2008) 205:1009–17. 10.1084/jem.20072457 18426987 PMC2373834

[83] Boeras DI Hraber PT Hurlston M Evans-Strickfaden T Bhattacharya T Giorgi EE Role of donor genital tract HIV-1 diversity in the transmission bottleneck. Proc Natl Acad Sci U S A (2011) 108:E1156–63. 10.1073/pnas.1103764108 22065783 PMC3219102

[84] Prince JL Claiborne DT Carlson JM Schaefer M Yu T Lahki S Role of transmitted Gag CTL polymorphisms in defining replicative capacity and early HIV-1 pathogenesis. PLoS Pathog (2012) 8:e1003041. 10.1371/journal.ppat.1003041 23209412 PMC3510241

[85] Yue L Pfafferott KJ Baalwa J Conrod K Dong CC Chui C Transmitted virus fitness and host T cell responses collectively define divergent infection outcomes in two HIV-1 recipients. PLoS Pathog (2015) 11:e1004565. 10.1371/journal.ppat.1004565 25569444 PMC4287535

[86] Mphatswe W Blanckenberg N Tudor-Williams G Prendergast A Thobakgale C Mkhwanazi N High frequency of rapid immunological progression in African infants infected in the era of perinatal HIV prophylaxis. AIDS (2007) 21:1253–61. 10.1097/QAD.0b013e3281a3bec2 17545701

[87] Mori M Adland E Paioni P Swordy A Mori L Laker L Sex differences in antiretroviral therapy initiation in pediatric HIV infection. PLoS One (2015) 10:e0131591. 10.1371/journal.pone.0131591 26151555 PMC4494714

[88] Muenchhoff M Prendergast AJ Goulder PJ Immunity to HIV in early life. Front Immunol (2014) 5:391 10.3389/fimmu.2014.00391 25161656 PMC4130105

[89] Feeney ME Tang Y Roosevelt KA Leslie AJ Mcintosh K Karthas N Immune escape precedes breakthrough human immunodeficiency virus type 1 viremia and broadening of the cytotoxic T-lymphocyte response in an HLA-B27-positive long-term-nonprogressing child. J Virol (2004) 78:8927–30. 10.1128/JVI.78.16.8927-8930.2004 15280502 PMC479057

[90] Liu D Zuo T Hora B Song H Kong W Yu X Preexisting compensatory amino acids compromise fitness costs of a HIV-1 T cell escape mutation. Retrovirology (2014) 11:101. 10.1186/s12977-014-0101-0 25407514 PMC4264250

[91] Feeney ME Tang Y Pfafferott K Roosevelt KA Draenert R Trocha A HIV-1 viral escape in infancy followed by emergence of a variant-specific CTL response. J Immunol (2005) 174:7524–30. 10.4049/jimmunol.174.12.7524 15944251

[92] Friedrich TC Dodds EJ Yant LJ Vojnov L Rudersdorf R Cullen C Reversion of CTL escape-variant immunodeficiency viruses in vivo. Nat Med (2004) 10:275–81. 10.1038/nm998 14966520

[93] Leslie A Kavanagh D Honeyborne I Pfafferott K Edwards C Pillay T Transmission and accumulation of CTL escape variants drive negative associations between HIV polymorphisms and HLA. J Exp Med (2005) 201:891–902. 10.1084/jem.20041455 15781581 PMC2213090

[94] Bhattacharya T Daniels M Heckerman D Foley B Frahm N Kadie C Founder effects in the assessment of HIV polymorphisms and HLA allele associations. Science (2007) 315:1583–6. 10.1126/science.1131528 17363674

[95] Matthews PC Koyanagi M Kloverpris HN Harndahl M Stryhn A Akahoshi T Differential clade-specific HLA-B*3501 association with HIV-1 disease outcome is linked to immunogenicity of a single Gag epitope. J Virol (2012) 86:12643–54. 10.1128/JVI.01381-12 22973023 PMC3497693

[96] Payne R Muenchhoff M Mann J Roberts HE Matthews P Adland E Impact of HLA-driven HIV adaptation on virulence in populations of high HIV seroprevalence. Proc Natl Acad Sci U S A (2014) 111:E5393–400. 10.1073/pnas.1413339111 25453107 PMC4273423

[97] Fryer HR Frater J Duda A Roberts MG Investigators ST Phillips RE Modelling the evolution and spread of HIV immune escape mutants. PLoS Pathog (2010) 6:e1001196. 10.1371/journal.ppat.1001196 21124991 PMC2987822

[98] Cotton LA Kuang XT Le AQ Carlson JM Chan B Chopera DR Genotypic and functional impact of HIV-1 adaptation to its host population during the North American epidemic. PLoS Genet (2014) 10:e1004295. 10.1371/journal.pgen.1004295 24762668 PMC3998893

[99] Loffredo JT Burwitz BJ Rakasz EG Spencer SP Stephany JJ Vela JP The antiviral efficacy of simian immunodeficiency virus-specific CD8+ T cells is unrelated to epitope specificity and is abrogated by viral escape. J Virol (2007) 81:2624–34. 10.1128/JVI.01912-06 17192314 PMC1866004

[100] Adland E Carlson JM Paioni P Kloverpris H Shapiro R Ogwu A Nef-specific CD8+ T cell responses contribute to HIV-1 immune control. PLoS One (2013) 8:e73117. 10.1371/journal.pone.0073117 24023819 PMC3759414

[101] Valentine LE Loffredo JT Bean AT Leon EJ Macnair CE Beal DR Infection with “escaped” virus variants impairs control of simian immunodeficiency virus SIVmac239 replication in Mamu-B*08-positive macaques. J Virol (2009) 83:11514–27. 10.1128/JVI.01298-09 19726517 PMC2772717

[102] Kloverpris HN Adland E Koyanagi M Stryhn A Harndahl M Matthews PC HIV subtype influences HLA-B*07:02-associated HIV disease outcome. AIDS Res Hum Retroviruses (2014) 30:468–75. 10.1089/AID.2013.0197 24010680 PMC4010166

[103] Kawashima Y Kuse N Gatanaga H Naruto T Fujiwara M Dohki S Long-term control of HIV-1 in hemophiliacs carrying slow-progressing allele HLA-B*5101. J Virol (2010) 84:7151–60. 10.1128/JVI.00171-10 20410273 PMC2898248

[104] Herbeck JT Muller V Maust BS Ledergerber B Torti C Di Giambenedetto S Is the virulence of HIV changing? A meta-analysis of trends in prognostic markers of HIV disease progression and transmission. AIDS (2012) 26:193–205. 10.1097/QAD.0b013e32834db418 22089381 PMC3597098

[105] Mellors JW Rinaldo CR Jr Gupta P White RM Todd JA Kingsley LA. Prognosis in HIV-1 infection predicted by the quantity of virus in plasma. Science (1996) 272:1167–70. 10.1126/science.272.5265.1167 8638160

[106] Seage GR III Holte S Gross M Koblin B Marmor M Mayer KH Case-crossover study of partner and situational factors for unprotected sex. J Acquir Immune Defic Syndr (2002) 31:432–9. 10.1097/00126334-200212010-00010 12447015

[107] Group I-ES Committee SS Abrams D Levy Y Losso MH Babiker A Interleukin-2 therapy in patients with HIV infection. N Engl J Med (2009) 361:1548–59. 10.1056/NEJMoa0903175 19828532 PMC2869083

[108] Giorgi JV Liu Z Hultin LE Cumberland WG Hennessey K Detels R. Elevated levels of CD38+ CD8+ T cells in HIV infection add to the prognostic value of low CD4+ T cell levels: results of 6 years of follow-up. The Los Angeles Center, Multicenter AIDS Cohort Study. J Acquir Immune Defic Syndr (1993) 6:904–12. 7686224

[109] Silvestri G Sodora DL Koup RA Paiardini M O’Neil SP Mcclure HM Nonpathogenic SIV infection of sooty mangabeys is characterized by limited bystander immunopathology despite chronic high-level viremia. Immunity (2003) 18:441–52. 10.1016/S1074-7613(03)00060-8 12648460

[110] Jacquelin B Mayau V Targat B Liovat AS Kunkel D Petitjean G Nonpathogenic SIV infection of African green monkeys induces a strong but rapidly controlled type I IFN response. J Clin Invest (2009) 119:3544–55. 10.1172/JCI40093 19959873 PMC2786805

[111] Deeks SG Kitchen CM Liu L Guo H Gascon R Narvaez AB Immune activation set point during early HIV infection predicts subsequent CD4+ T-cell changes independent of viral load. Blood (2004) 104:942–7. 10.1182/blood-2003-09-3333 15117761

[112] Hunt PW Sinclair E Rodriguez B Shive C Clagett B Funderburg N Gut epithelial barrier dysfunction and innate immune activation predict mortality in treated HIV infection. J Infect Dis (2014) 210:1228–38. 10.1093/infdis/jiu238 24755434 PMC4192038

[113] Bosinger SE Li Q Gordon SN Klatt NR Duan L Xu L Global genomic analysis reveals rapid control of a robust innate response in SIV-infected sooty mangabeys. J Clin Invest (2009) 119:3556–72. 10.1172/JCI40115 19959874 PMC2786806

[114] Goulder PJ Walker BD. HIV and HLA class I: an evolving relationship. Immunity (2012) 37:426–40. 10.1016/j.immuni.2012.09.005 22999948 PMC3966573

[115] Claiborne DT Prince JL Scully E Macharia G Micci L Lawson B Replicative fitness of transmitted HIV-1 drives acute immune activation, proviral load in memory CD4+ T cells, and disease progression. Proc Natl Acad Sci U S A (2015) 112:E1480–9. 10.1073/pnas.1421607112 25730868 PMC4378387

[116] Paiardini M Cervasi B Reyes-Aviles E Micci L Ortiz AM Chahroudi A Low levels of SIV infection in sooty mangabey central memory CD(4)(+) T cells are associated with limited CCR5 expression. Nat Med (2011) 17:830–6. 10.1038/nm.2395 21706028 PMC3253129

[117] Hecht FM Hartogensis W Bragg L Bacchetti P Atchison R Grant R HIV RNA level in early infection is predicted by viral load in the transmission source. AIDS (2010) 24:941–5. 10.1097/QAD.0b013e328337b12e 20168202 PMC2887742

[118] Brander C Walker BD. Gradual adaptation of HIV to human host populations: good or bad news? Nat Med (2003) 9:1359–62. 10.1038/nm941 14595431

[119] Campbell TB Schneider K Wrin T Petropoulos CJ Connick E. Relationship between in vitro human immunodeficiency virus type 1 replication rate and virus load in plasma. J Virol (2003) 77:12105–12. 10.1128/JVI.77.22.12105-12112.2003 14581547 PMC253754

[120] Brockman MA Brumme ZL Brumme CJ Miura T Sela J Rosato PC Early selection in Gag by protective HLA alleles contributes to reduced HIV-1 replication capacity that may be largely compensated for in chronic infection. J Virol (2010) 84:11937–49. 10.1128/JVI.01086-10 20810731 PMC2977869

[121] Brumme ZL Li C Miura T Sela J Rosato PC Brumme CJ Reduced replication capacity of NL4-3 recombinant viruses encoding reverse transcriptase-integrase sequences from HIV-1 elite controllers. J Acquir Immune Defic Syndr (2011) 56:100–8. 10.1097/QAI.0b013e3181fe9450 21124229 PMC3078702

[122] Nomura S Hosoya N Brumme ZL Brockman MA Kikuchi T Koga M Significant reductions in Gag-protease-mediated HIV-1 replication capacity during the course of the epidemic in Japan. J Virol (2013) 87:1465–76. 10.1128/JVI.02122-12 23152532 PMC3554148

[123] Juarez-Molina CI Payne R Soto-Nava M Avila-Rios S Valenzuela-Ponce H Adland E Impact of HLA selection pressure on HIV fitness at a population level in Mexico and Barbados. J Virol (2014) 88:10392–8. 10.1128/JVI.01162-14 25008926 PMC4178877

[124] Fraser C Hollingsworth TD Chapman R De Wolf F Hanage WP. Variation in HIV-1 set-point viral load: epidemiological analysis and an evolutionary hypothesis. Proc Natl Acad Sci U S A (2007) 104:17441–6. 10.1073/pnas.0708559104 17954909 PMC2077275

[125] Pantazis N Porter K Costagliola D De Luca A Ghosn J Guiguet M Temporal trends in prognostic markers of HIV-1 virulence and transmissibility: an observational cohort study. Lancet HIV (2014) 3:119–26. 10.1016/S2352-3018(14)00002-2 26424120

[126] Adland E Klenerman P Goulder P Matthews PC Ongoing burden of disease and mortality from HIV/CMV coinfection in Africa in the antiretroviral therapy era. Front Microbiol (2015) 6:1016 10.3389/fmicb.2015.01016 26441939 PMC4585099

[127] Gao F Bailes E Robertson DL Chen Y Rodenburg CM Michael SF Origin of HIV-1 in the chimpanzee Pan troglodytes troglodytes. Nature (1999) 397:436–41. 10.1038/17130 9989410

[128] Gray RH Wawer MJ Brookmeyer R Sewankambo NK Serwadda D Wabwire-Mangen F Probability of HIV-1 transmission per coital act in monogamous, heterosexual, HIV-1-discordant couples in Rakai, Uganda. Lancet (2001) 357:1149–53. 10.1016/S0140-6736(00)04331-2 11323041

[129] Wawer MJ Reynolds SJ Serwadda D Kigozi G Kiwanuka N Gray RH Might male circumcision be more protective against HIV in the highly exposed? An immunological hypothesis. AIDS (2005) 19:2181–2. 10.1097/01.aids.0000194132.51006.4f 16284475

[130] Powers KA Poole C Pettifor AE Cohen MS. Rethinking the heterosexual infectivity of HIV-1: a systematic review and meta-analysis. Lancet Infect Dis (2008) 8:553–63. 10.1016/S1473-3099(08)70156-7 18684670 PMC2744983

[131] Baggaley RF White RG Boily MC. HIV transmission risk through anal intercourse: systematic review, meta-analysis and implications for HIV prevention. Int J Epidemiol (2010) 39:1048–63. 10.1093/ije/dyq057 20406794 PMC2929353

[132] Goodreau SM Cassels S Kasprzyk D Montano DE Greek A Morris M. Concurrent partnerships, acute infection and HIV epidemic dynamics among young adults in Zimbabwe. AIDS Behav (2012) 16:312–22. 10.1007/s10461-010-9858-x 21190074 PMC3394592

[133] Dennis AM Herbeck JT Brown AL Kellam P De Oliveira T Pillay D Phylogenetic studies of transmission dynamics in generalized HIV epidemics: an essential tool where the burden is greatest? J Acquir Immune Defic Syndr (2014) 67:181–95. 10.1097/QAI.0000000000000271 24977473 PMC4304655

[134] Hollingsworth TD Pilcher CD Hecht FM Deeks SG Fraser C. High transmissibility during early HIV infection among men who have sex with men-San Francisco, California. J Infect Dis (2015) 211:1757–60. 10.1093/infdis/jiu831 25542958 PMC4425938

